# Central modulation of parasympathetic outflow is impaired in de novo Parkinson's disease patients

**DOI:** 10.1371/journal.pone.0210324

**Published:** 2019-01-17

**Authors:** Carlo Tessa, Nicola Toschi, Stefano Orsolini, Gaetano Valenza, Claudio Lucetti, Riccardo Barbieri, Stefano Diciotti

**Affiliations:** 1 Department of Radiology and Nuclear Medicine, Versilia Hospital, Azienda USL Toscana Nord Ovest, Lido di Camaiore (Lu), Italy; 2 Medical Physics Section, Department of Biomedicine and Prevention, University of Rome “Tor Vergata”, Rome, Italy; 3 Department of Radiology, Athinoula A. Martinos Center for Biomedical Imaging and Harvard Medical School, Massachusetts General Hospital, Boston, MA, United States of America; 4 Department of Electrical, Electronic, and Information Engineering “Guglielmo Marconi”, University of Bologna, Cesena, Italy; 5 Department of Anesthesia, Massachusetts General Hospital, Boston, MA, United States of America; 6 Research Center E. Piaggio and Department of Information Engineering, School of Engineering, University of Pisa, Pisa, Italy; 7 Division of Neurology, Versilia Hospital, Azienda USL Toscana Nord Ovest, Lido di Camaiore (Lu), Italy; 8 Department of Electronics, Informatics and Bioengineering, Politecnico di Milano, Milano, Italy; Heidelberg University, GERMANY

## Abstract

Task- and stimulus-based neuroimaging studies have begun to unveil the central autonomic network which modulates autonomic nervous system activity. In the present study, we aimed to evaluate the central autonomic network without the bias constituted by the use of a task. Additionally, we assessed whether this circuitry presents signs of dysregulation in the early stages of Parkinson’s disease (PD), a condition which may be associated with dysautonomia. We combined heart-rate-variability based methods for time-varying assessments of the autonomic nervous system outflow with resting-state fMRI in 14 healthy controls and 14 de novo PD patients, evaluating the correlations between fMRI time-series and the instantaneous high-frequency component of the heart-rate-variability power spectrum, a marker of parasympathetic outflow. In control subjects, the high-frequency component of the heart-rate-variability power spectrum was significantly anti-correlated with fMRI time-series in several cortical, subcortical and brainstem regions. This complex central network was not detectable in PD patients. In between-group analysis, we found that in healthy controls the brain activation related to the high-frequency component of the heart-rate-variability power spectrum was significantly less than in PD patients in the mid and anterior cingulum, sensorimotor cortex and supplementary motor area, insula and temporal lobe, prefrontal cortex, hippocampus and in a region encompassing posterior cingulum, precuneus and parieto-occipital cortex. Our results indicate that the complex central network which modulates parasympathetic outflow in the resting state is impaired in the early clinical stages of PD.

## Introduction

While the brainstem centers that regulate the autonomic nervous system (ANS) are well known from animal studies, less is known about the cerebral regions involved in central control of ANS functions in humans. Still, animal studies have converged towards proposing the existence of a central autonomic network (CAN) that encompasses the insula, medial prefrontal cortex, cingulum, thalamus and amygdala and is involved in integrating and regulating autonomic function [1]. In more recent years, non-invasive neuroimaging methods have allowed to evaluate central autonomic centers in humans by combining the analysis of autonomic outflow metrics like heart-rate-variability (HRV) (defined through analysis of the time intervals between two consecutive R waves in the electrocardiogram (ECG)), with Positron Emission Tomography (PET) or functional magnetic resonance imaging (fMRI). These studies have highlighted that the CAN is more extended than previously hypothesized, possibly involving a number of cortical, subcortical and cerebellar regions (for reviews, see [2, 3]).

In these studies, HRV changes have been correlated with brain fMRI/PET responses during several tasks and stimuli that are known to involve the ANS (for reviews, see [2, 3]). However, the results of such studies are selectively dependent on the type of the task or stimulus employed. Therefore, a task and stimulus-free approach to CAN evaluation is desirable. To the best of our knowledge, only one previous seed-based work [4] has investigated functional connectivity in conjunction with HRV in the resting state. In this study, which was focused on connectivity of the dorsal anterior cingulate cortex and amygdale in normal subjects, a number of cortical region have been found to increase their functional connectivity with the seed regions during states of elevated HRV.

Symptoms of cardiovascular dysautonomia are common in Parkinson’s disease (PD) and have a significant impact on quality of life and daily activities of PD patients [5, 6]. While dysautonomia is more frequent in the advanced stages of the disease, it can also occur in de novo (drug naïve) patients [7, 8]. This is in agreement with studies in PD patients that have demonstrated the presence of Lewy bodies and degenerative changes in various autonomic regulatory regions [9–12].

Previous studies have also described HRV changes in PD, both in advanced disease stages [13–15] and in de novo patients [16, 17]. In this context, using a probabilistic point-process framework, dynamical HRV measures [18] able to discriminate subtle ANS dysfunction in PD have been recently developed [19–21].

In the present study, we aimed at a) performing a whole brain evaluation of the neural correlates of ANS modulation without the bias constituted by the use of a task or stimulus or the constraint of a seed-based analysis, and b) assessing whether this brain circuitry is dysregulated in the early stages of PD, when patients are free of treatment-related confounds in ANS function. To this purpose, we have combined, for the first time, dynamical HRV-based methods for time-varying assessments of ANS outflow with resting-state fMRI in 14 healthy controls and 14 de novo (drug naïve) PD patients, and have evaluated the correlations between fMRI time-series and the instantaneous high-frequency (0.15–0.50 Hz) component of the HRV power spectrum (HF-HRV), a parasympathetic metric [22].

## Materials and methods

### Subjects

The Ethics Committee of Area Vasta Nord Ovest (CEAVNO) approved this research. Fourteen (3 women and 11 men, age 63.7±11.1 years, mean ± standard deviation) patients with de-novo parkinsonian syndrome consecutively referred to a Neurology Unit for the evaluation of PD over a 24-month interval (from June 2012 to June 2014) were recruited in this prospective study. Clinical evaluation included history of disease-related symptoms and signs and neurological examination. All subjects were clinically screened for cardiovascular and gastrointestinal symptoms, urinary and sexual dysfunction, thermoregulatory changes and pupillary abnormalities. Signs and symptoms of autonomic dysfunction suspicious of atypical Parkinsonism were considered exclusion criteria. All patients satisfied the criteria for the diagnosis of PD of the UK Parkinson’s Disease Society Brain Bank [23]. Severity of the disease was evaluated by the Hoehn–Yahr (HY) ranking scale [24] and the Unified Parkinson's Disease Rating Scale (UPDRS) [25]. PD patients were screened for depressive features by the Geriatric Depression Scale Short Form [26]. Diagnosis of depression was made according to DSM IV-TR (Diagnostic and Statistical Manual of Mental Disorders, Fourth Edition, Text-Revision) criteria [27]. A 123IFP-CIT SPECT scan was performed to confirm nigrostriatal degeneration. After clinical evaluation, all patients underwent MRI examination; then, the dopaminergic treatment was started. Patients were clinically assessed every six months. The follow-up was at least one year [mean ± standard deviation (SD), 2.4 ± 0.4 years] in order to evaluate the treatment response and the appearance of signs of atypical parkinsonism.

Fourteen age- and gender-matched healthy subjects (3 women and 11 men, age 64.7±9.6 years, mean ± SD) with no history of neurological diseases and normal neurological examination were recruited as controls. No significant difference in age was found between the two groups (p = 0.93, Mann-Whitney test, null-hypothesis of equal medians) and the same proportion of gender was observed.

None of the patients and controls had any disease (except PD in the patient group) or took drugs known to affect the autonomic nervous system. All subjects gave their written informed consent to participate in the study. The clinical features of PD patients are detailed in S1 Table.

### MRI examination and physiological monitoring

MRI was performed on a 1.5 T MR scanner system (Magnetom Avanto, software version Syngo MR B17, Siemens, Erlangen-Germany) equipped with a 12-element matrix radiofrequency head coil and SQ-engine gradients. The SQ-engine gradients had maximum strength of 45 mT/m and slew rate of 200 T/m/s.

All subjects underwent high resolution 3D T1-weighted imaging and resting state fMRI (rsfMRI), the latter with simultaneous cardiorespiratory monitoring. T1-weighted MR images were acquired with an axial high resolution 3D sequence (Magnetization Prepared Rapid Gradient Echo, MPRAGE) with repetition time (TR) = 1900 ms, echo time (TE) = 3.44 ms, inversion time (TI) = 1100 ms, flip angle = 15°, slice thickness = 0.86 mm, field of view (FOV) = 220 mm×220 mm, matrix size = 256×256, number of excitations (NEX) = 2. A fluid attenuated inversion recovery (FLAIR) sequence (TR = 9000 ms, TE = 88 ms, TI = 2500 ms, slice thickness = 3 mm, FOV = 172.5 mm × 230 mm, matrix size = 154×256, turbo factor = 16, NEX = 1) was also obtained in the axial plane. For the rsfMRI experiments, we used a T2*-weighted echo-planar imaging (EPI) sequence (TR = 2130 ms, TE = 40 ms, flip angle = 90°, slice thickness = 5 mm; FOV = 256 mm×256 mm, matrix size 64×64; number of slices = 32; interleaved slice acquisition) exploiting the blood-oxygen-level-dependent (BOLD) effect. Two hundred and thirty volumes were acquired for a total acquisition time of about 8 minutes and 10 seconds. The slices were oriented along and parallel to the bi-commissural plane and covered the entire brain. During rsfMRI acquisition the subjects were instructed to lie still with their eyes closed and not to think of anything in particular. Cushions were used to minimize head motion during the scan.

Physiological signals (pulse oximetry and respiratory signals) for HRV assessment were recorded simultaneously with the rsfMRI examination through a built-in Siemens physiological measurement unit. Patients were instrumented with a peripheral pulse sensor on the left hand index finger and a respiratory cushion in contact with the upper abdomen attached to the patient via a respiratory belt. Pulse and respiratory signals were sampled at 50 Hz. In order to provide enough signal time for initializing the point process model (see "Dynamical HRV assessment" section below), physiological recordings were started 2 minutes before the beginning of the rsfMRI sequence.

Synchronization of DICOM images with physiological recordings was carried out by using time-stamps derived from the scanner clock, stored both in DICOM images and in physiological log files.

### Data analysis

**Fig 1:**
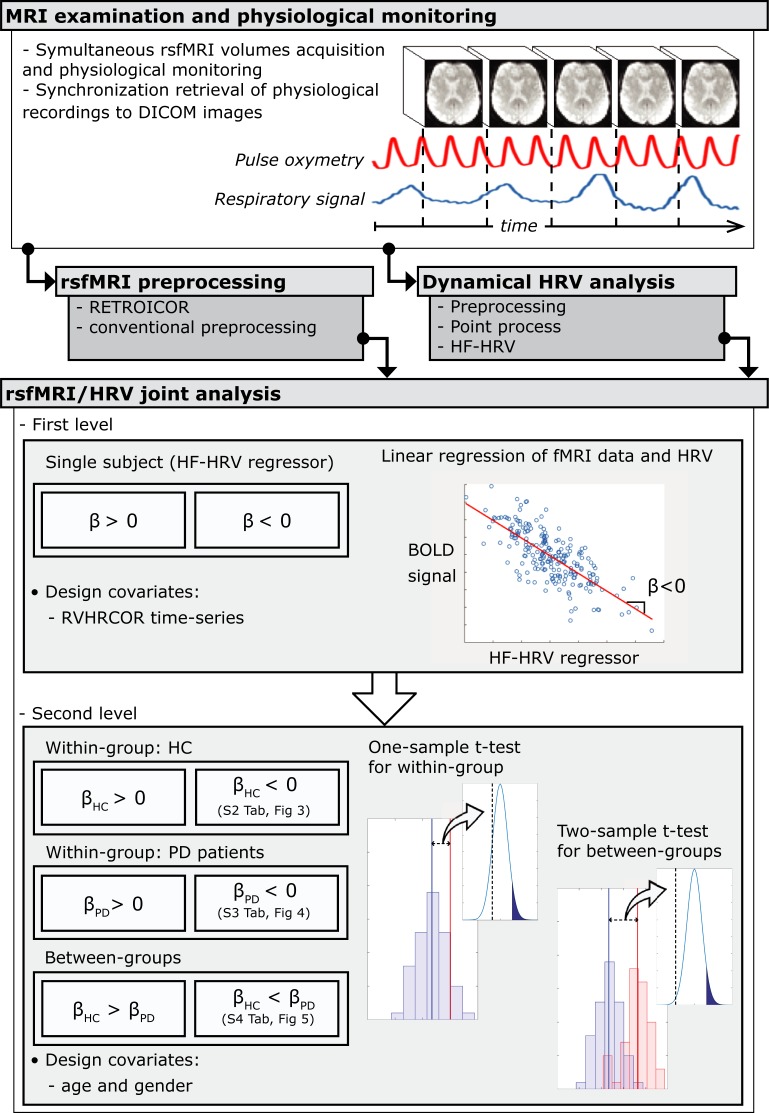
Architectural diagram of data analysis. Resting-state fMRI and physiological signals have been acquired simultaneously: a separate pre-processing is followed by a joint rsfMRI/HRV analysis. β is the effect of HF-HRV while controlling for RVHRCOR regressors in the first level GLM. β_HC_ and β_PD_ are the mean brain activity relating to HF-HRV regressor while controlling for age and gender in the healthy controls (HC) and PD patients group, respectively.

Physiological signals and MRI images were preprocessed separately and successively entered into joint rsfMRI-HRV analysis (see below and Fig 1).

#### Dynamical HRV assessment

R–R intervals were obtained annotating pulse data through automated methods [28]. The absence of detection errors or ectopic beats was ensured by the application of an effective point process-based artifact removal method [28], as well as visual inspection. Through this approach, over all records, 99.9282% of total beats were retained for further analyses (i.e., 55 beats were corrected).

We then applied a linear point-process method [18] to compute instantaneous estimates of heart rate (HR) and HRV defined in the time and frequency domains. Briefly, this approach models the stochastic nature of heartbeat generation considering a physiologically plausible, history-dependent, inverse-Gaussian process of ventricular repolarization [18]. More specifically, the mean value of the inverse-Gaussian probability function explains the dependence on past R–R intervals as a linear function of the past k samples. This allows us to use the k regressive coefficients to estimate the dynamics of the sympathetic and vagal influences on the sinoatrial node of the heart, as well as the total spectral power as decomposed in the high frequency (HF, 0.15–0.5 Hz) spectral component. Of note, all the time-varying HRV measures can be estimated at arbitrary time resolution, and can be therefore accurately aligned to fMRI volumes in time.

In this study, as a reference measure of HR we have considered the instantaneous HR index, i.e., the mean of the inverse-Gaussian function associated with the inverse variable of the R-waves time occurrences. The instantaneous HF-HRV index has been considered to estimate the vagal influence on the sinus node. Both HR and HF-HRV series were estimated by a fixed model with a regression of order k = 8, determined after preliminary goodness-of-fit analysis of the data, and were updated every Δ = 5 ms. In order to be synchronized with the fMRI TR series, the series were further low-pass filtered and resampled at the proper time points. Before being used as regressors in the fMRI analysis, the HF-HRV power was filtered using a 12-span local linear regression with weighted linear least squares. Subject specific median (across time) HF-HRV values were compared group-wise using nonparametric statistics (Mann-Whitney U test).

#### Custom T1-weighted template construction

In order to improve co-registration accuracy within our specific population, we build a custom T1 template specific to this study. This involved using all T1-weighted images which, using the ANTs package [29], were co-registered and averaged iteratively [30], where the group average was recreated at the end of each iteration. The procedure was based on symmetrical diffeomorphic mapping (and in particular the SyN tool). In this study, we employed five total iterations [31, 32].

#### RsfMRI pre-processing

We analyzed rsFMRI data were analyzed in FEAT (FMRI Expert Analysis Tool), version 6.00, part of FSL (FMRIB's Software Library, www.fmrib.ox.ac.uk/fsl) and AFNI, (latest release on July 2nd 2014). Preprocessing involved: (1) removal of the first 5 dummy scans; (2) regressing-out of cardiac and respiratory artifacts by using retrospective image correction (RETROICOR) [33]; (3) motion correction using MCFLIRT [the mean (across voxels) absolute displacement (each time point with respect to the middle-time point rsfMRI image) was less than 1.15 mm, i.e. less than half a voxel] [34]; (4) slice-timing correction using Fourier-space time-series phase-shifting; (5) high-pass temporal filtering (Gaussian-weighted least squares straight line fitting) with cut-off of 100 s; (6) spatial smoothing (10 mm FWHM); (7) grand-mean intensity normalization with no additional temporal low-pass filtering [35].

RsfMRI images were then co-registered to the custom T1 template through affine boundary-based registration (BBR) [36] in FLIRT. The individual T1-weighted images were co-registered to the custom T1-weighted template using an affine transformation [34] which was then further refined using non-linear transformations in FNIRT [37]. All above transformations were concatenated into a single warp which took subject-specific rsfMRI images into the custom template space for statistical analysis (see below) in a single step. Additionally, we co-registered the custom template to the standard-space Montreal Neurological Institute (MNI) 152 brain using an affine and non-linear transformation [37]. These transformations were used for atlas-based localization purposes of statistically significant clusters (see below).

#### Joint RsfMRI/HRV analysis

**Fig 2:**
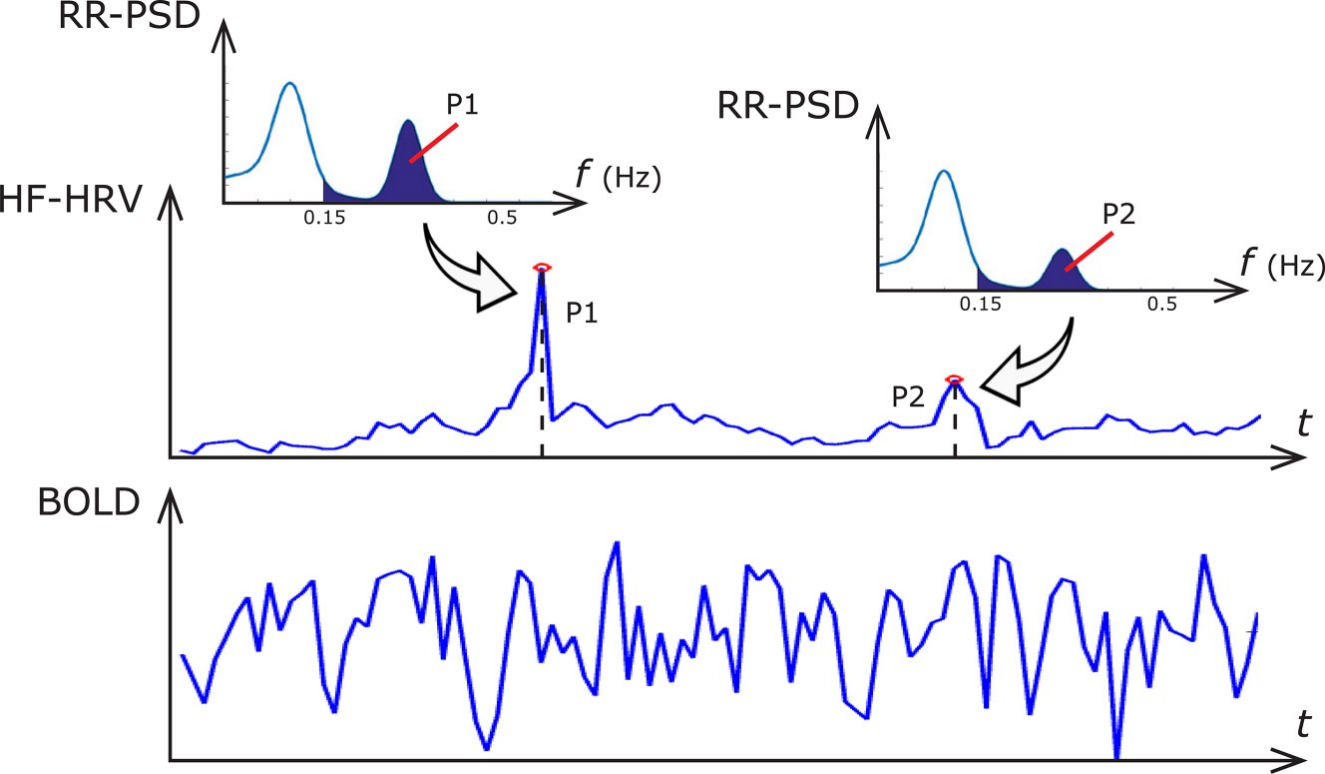
Illustration of the significance of dynamic HF-HRV estimate. At each point in time, the value of the HF-HRV signal (top pane) is equivalent to the power spectral density of the RR series (RR-PSD) which, in a classical approach, is calculated by integrating the Fourier-derived RR-PSD in the HF band (depicted in insets). The dynamic HF-HRV signal is synchronous with the measured BOLD signal (bottom pane).

To establish the neural correlates of ANS in every subject, we used time-resolved estimates of parasympathetic outflow (HF-HRV) as an explanatory variable in first-level analysis (Fig 2). Additional physiological noise was modeled using combined respiratory variation and heart rate correction (RVHRCOR) [35, 38] as nuisance covariates. The respiratory variation (RV) time series was calculated as the standard deviation of the respiratory signal over a 6s sliding window centered on each TR [35], whereas the HR time series were calculated from the RR series extracted above as the inverse of the average beat-to-beat interval in a 6s sliding window [35]. The HF-HRV regressor was formed by convolving the HF-HRV time-series with a double gamma hemodynamic response function. We tested both positive and negative correlations between rsfMRI time-series and HF-HRV regressor within the same general linear model (GLM).

To then test the hypothesis of group-wise correlations (as above) both in healthy subjects and in PD, we employed one-sample t-test and a mixed effects model implemented in FLAME (FMRIB’s Local Analysis of Mixed Effects) [39], including age and gender as nuisance covariates.

Additionally, we compared (healthy controls vs. PD patients), i.e. the regression coefficients (β's) from first-level analysis. This was accomplished by between-group analysis using unpaired t-test and a mixed effects model using FLAME [39], again including age and gender as nuisance covariates.

In each analysis, Z (Gaussianized T/F) statistic images were thresholded using clusters determined by Z>2.3 value and a (corrected) cluster significance threshold of p<0.05 [40].

Finally, we reported in-sample effect size of our primary outcome, i.e. of the between-group analysis, following the American Psychologist Association (APA) guidelines [41]. Given that unstandardized effect size (i.e. the difference between regression coefficients fitted while controlling for nuisance covariates) could be cumbersome to interpret, we chose to report a standardized voxel-wise effect size through Cohen’s d [42].

All group analyses were performed in the custom T1-weighted template space and the resulting thresholded Z statistic images were transformed into MNI space by applying the affine transform and the nonlinear warp described above. Spatial localization of significant clusters at group analyses was performed using the automated anatomical labeling (AAL) atlas [43].

## Results

### Within-group analyses

#### Healthy control group

**Fig 3:**
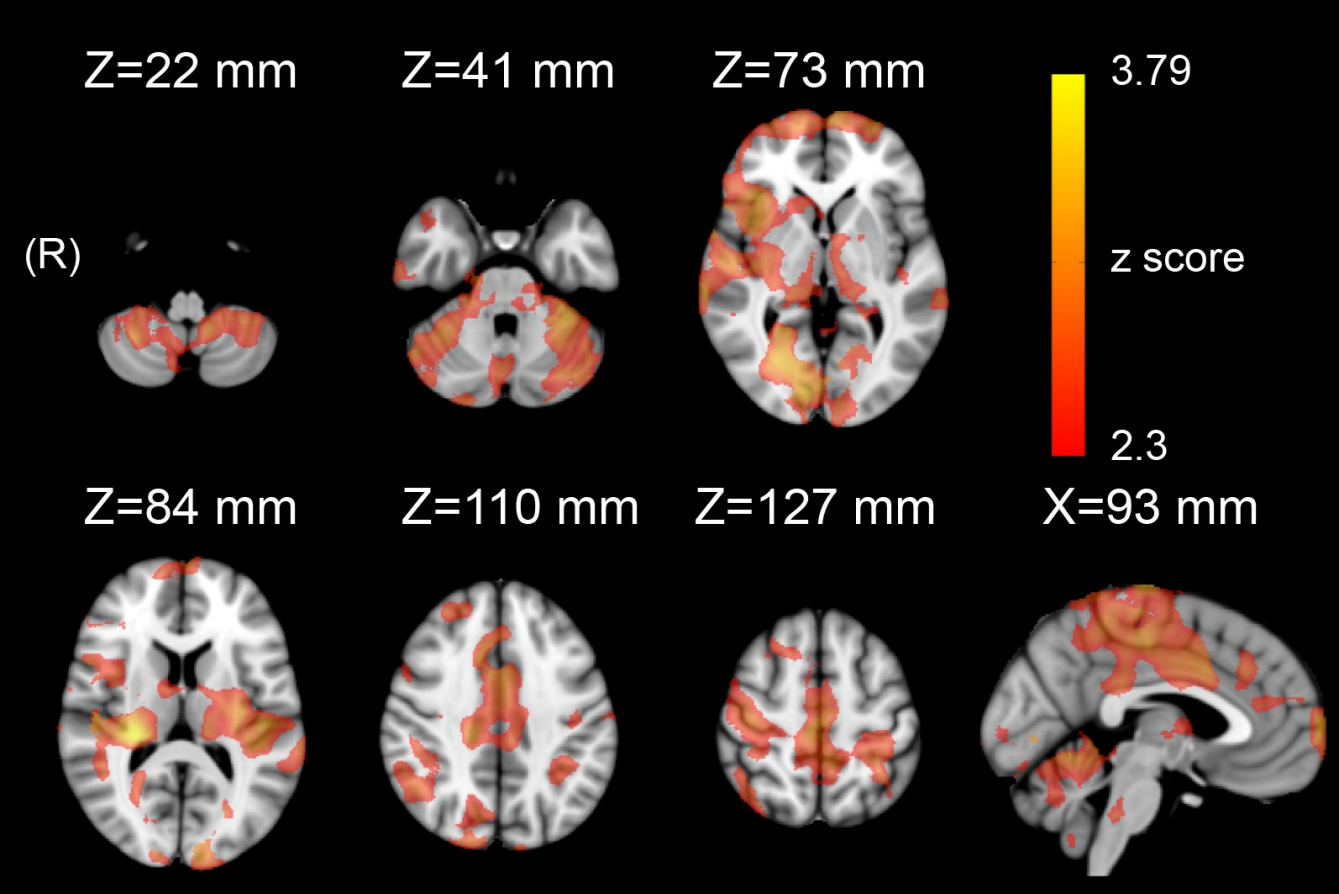
Statistical map (Z>2.3, p<0.05 corrected) showing that brain activation is significantly anti-correlated to HRV-based assessment of parasympathetic outflow (HF-HRV) in a group of 14 healthy controls.

We found that HF-HRV was significantly anti-correlated with rsfMRI time-series in several areas (S2 Table and Fig 3), encompassing, bilaterally, anterior, middle and posterior cingulum, insula, sensorimotor cortex, supplementary motor area (SMA), hippocampus and parahippocampal cortex, basal ganglia, pons, cerebellar vermis and hemispheres as well as a number of cortical gyri (S2 Table). No significant positive correlations were observed.

#### De novo PD group

**Fig 4:**
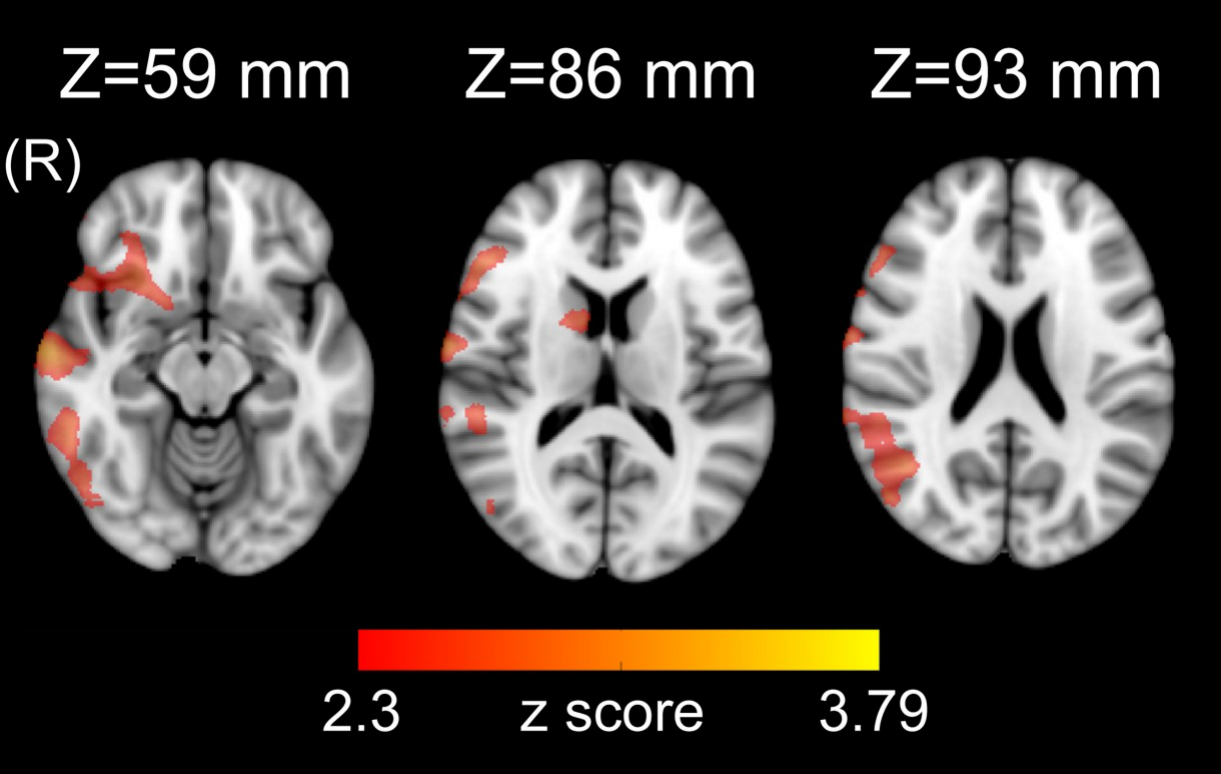
Statistical map (Z>2.3, p<0.05 corrected) showing that brain activation is significantly anti-correlated to HRV-based assessment of parasympathetic outflow (HF-HRV) in a group of 14 de novo PD patients.

We found that HF-HRV was significantly anti-correlated with rsfMRI time-series in right temporal superior, middle and inferior gyri, temporal pole, frontal middle, inferior and middle orbital gyri, olfactory gyrus, angular and supramarginal gyrus, Rolandic operculum, occipital inferior gyrus, caudate, putamen and pallidum (S3 Table and Fig 4). No significant positive correlations were observed.

### Between-group analysis

**Fig 5:**
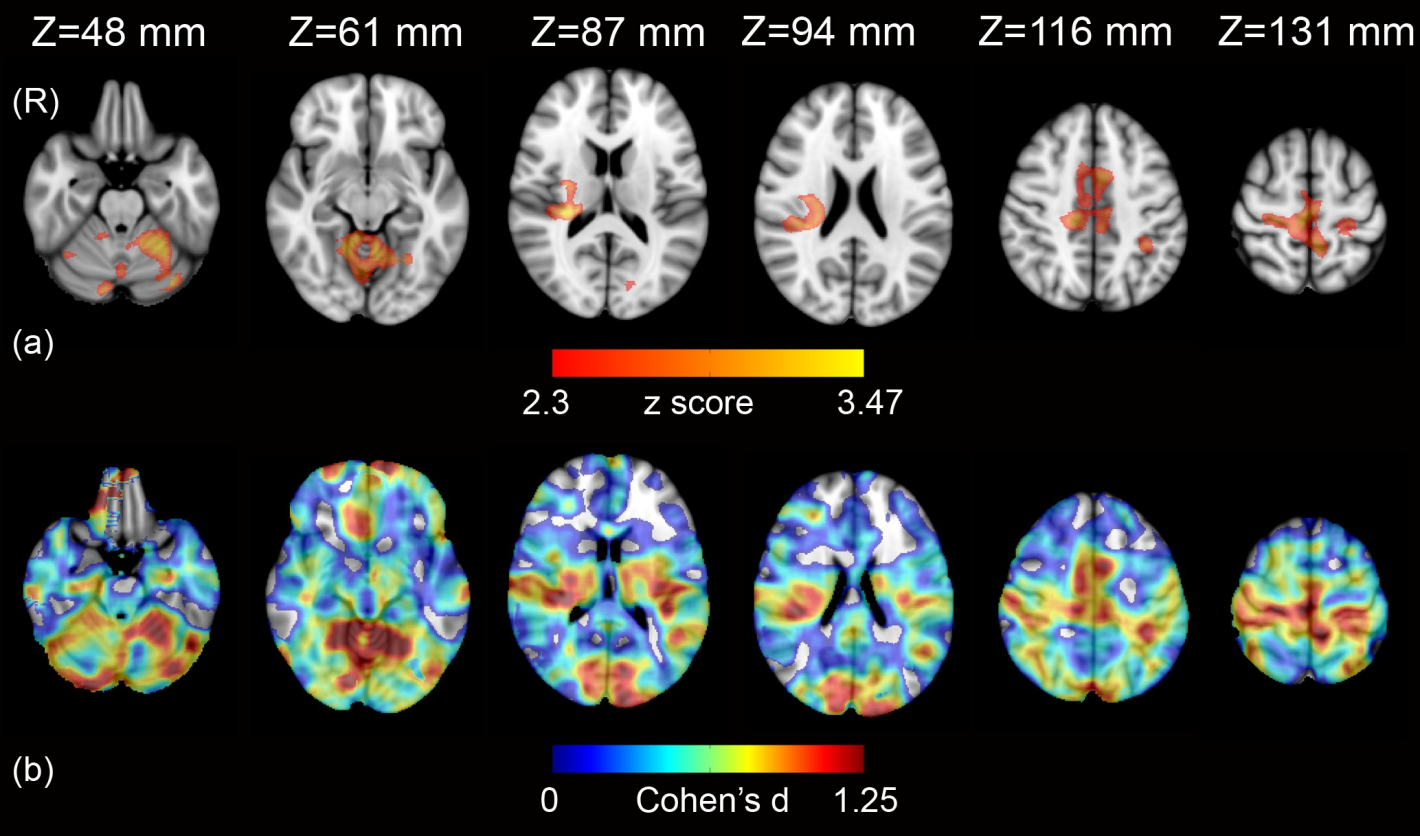
Statistical map (Z>2.3, p<0.05 corrected) showing areas where brain activation relating to HR-HRV in 14 healthy controls was significantly less than in 14 de novo PD patients (a), and effect size map (Cohen’s d>0) for the same axial slices (b).

In healthy controls, the brain activation related to HF-HRV was significantly less than in de novo PD patients in cerebellar vermis and hemispheres, bilateral middle cingulum, anterior left cingulum, bilateral sensorimotor cortex, bilateral SMA, right insula, right putamen, bilateral precuneus, left posterior cingulum, left parietal inferior gyrus, right supramarginal gyrus, left calcarine gyrus, bilateral lingual and fusiform gyrus, right temporal superior gyrus and Heschl gyrus, right frontal superior gyrus and right hippocampus (S4 Table and Fig 5).

## Discussion

### Neural correlates of ANS in healthy subjects

By combining a point-process framework for dynamical HRV assessment with fMRI analysis, we found that in control subjects several cortical, subcortical, cerebellar and brainstem regions modulate cardiovascular parasympathetic outflow in the resting state.

Previous studies in animal and human have consistently indicated the vast majority of these areas as constituents of the CAN [2, 3, 44]. Other regions, however, and in particular the occipital cortex, have been less frequently reported in this context [2] and their involvement in HRV modulation might be a peculiarity of the resting state condition.

Interestingly, both in healthy controls and PD patients, we consistently found an anti-correlation between rsfMRI time-series and HF-HRV in every part of the network of brain areas significantly associated with ANS activity. This could suggest that in the resting state, when parasympathetic (“rest and relax”) activity dominates over sympathetic (“fight or flight”) autonomic responses, a physiological decrease of brain activity in the CNS areas related to HRV modulation takes place, possibly reflecting lower physiological requests.

### Neural correlates of ANS dysfunction in PD

The anti-correlations between rsfMRI time-series and HF-HRV were weaker in PD patients. This differential behavior of the CAN in PD patients as compared to controls could represent either a compensatory attempt to maintain homeostasis, or an *a priori* pathologic functional activity, or both. Our results suggest that a disarrangement of central autonomic control, independent of treatment, already occurs in the early clinical stages of the disease.

To the best of our knowledge, the present study is the first to directly demonstrate changes in the CAN regulating cardiovagal outflow in PD. Comparing group differences in the correlations between fMRI time-series and the instantaneous high-frequency component of the HRV power spectrum (HF-HRV), we detected significant differences in mid and anterior cingulum, sensorimotor cortex and SMA, insula and temporal lobe, prefrontal cortex, hippocampus and in a region encompassing posterior cingulum, precuneus and adjacent parieto-occipital cortex.

The insula, anterior and mid cingulate cortex are key nodes of the CAN. In particular, the insula, regarded as the ‘visceral sensory’ cortex, is a somatotopically organized region that receives visceral sensory information and modulates ANS responses [2, 44], while the anterior and mid-cingulate cortex have been constantly associated with cardiovascular control [2, 3].

The link between the sensorimotor cortex and adjacent perimotor cortex as well as SMA and ANS modulation has been underlined in a recent meta-analysis of task-based imaging studies [2]. In the context of our resting state study, the anti-correlation between fMRI time-series in cortical motor areas and HF-HRV could reflect the decreased activation of areas linked to “fight or flight reaction” at rest, when parasympathetic influence predominates.

The possible role of the putamen in ANS modulation of cardiovascular and respiratory functions has been suggested both by experimental studies in animals and by imaging studies in humans [44–46]. Furthermore, putaminal involvement in pathological conditions with dysautonomia, such as Multiple Systems Atrophy, is well known [47].

The cerebellum plays an important role in autonomic cardiovascular control [48, 49]. Studies in animals have demonstrated the involvement of the cerebellum in blood pressure and heart rate modulation [48, 50] while several imaging studies have consistently reported cerebellar involvement in responses to tasks and stimuli that are known to cause HRV changes [51, 52].

Animal studies have evidenced the neural connections that link hippocampus and brainstem autonomic nuclei [53] as well as the cardiovascular responses elicited by hippocampal stimulation [54], while fMRI studies have shown the role of hippocampus in hearth rate modulation [2, 52].

Several imaging studies have underlined the role of precuneus/posterior cingulate cortex in ANS modulation [2, 55, 56]. The posteromedial parietal cortex is a key component of the default mode network [57], the task-negative network of associated brain regions that exhibits high levels of neural activity when an individual is at rest. Interestingly, in two previous studies in normal subjects, BOLD signal at rest in posterior cingulate and precuneus was found to positively correlate with concurrently recorded muscle sympathetic nerve activity (MSNA) [55] and skin sympathetic nerve activity (SSNA) [56], both metrics of ANS sympathetic outflow.

We also found significant differences between de novo PD patients and healthy controls in brain regions that have not been investigated in animal studies evaluating the CAN, and specifically in temporal superior, supramarginal and parietal inferior gyri, in lateral prefrontal cortex and parieto-occipital cortex. These regions, however, have been reported to be involved in ANS modulation in imaging studies (for reviews, see [2, 44]) and their role should be further addressed in the future. Our results reinforce the idea that the human CAN encompasses more regions than previously hypothesized.

We did not find significant activation related to HF-HRV in the amygdala, neither in healthy controls nor in PD patients. This was an unexpected result, given that the amygdala is considered a core constituent of the CAN [2–4, 44]. However, the amygdala has been shown to play a major role in the mediation of autonomic responses during emotionally relevant stimulation, and it has been suggested that amygdala may be a detector of potential threats, and a mediator of adaptive “fear” responses [3]. We therefore submit that during a resting state experiment, in which subjects are instructed to relax, the role of the amygdala in modulating HRV might be less relevant.

In healthy controls, we found a significant association between HF-HRV and BOLD signal in the dorsal pons, while we did not find significant autonomic-associated activation neither in the medulla nor in the mesencephalon. Indeed, according to Braak’s pathologic staging system for PD, the parasympathetic preganglionic neurons of the dorsal motor nucleus of the vagus in the medulla as well as the peripheral autonomic nerves and ganglia are affected by Lewy pathology and by neurodegeneration since the pre-symptomatic stages of the disease, and their involvement antedates that of cortical and subcortical structures [9, 58]. However, functional neuroimaging of the brainstem is problematic, because of artifacts due to blood and cerebrospinal fluid (CSF) pulsatility as well as to its close proximity with bone and air-filled cavities [59–61]. It is therefore possible that brainstem-focused approaches, probably encompassing higher field strength, smaller voxel size and tailored co-registration methods [62], could aid in unveiling autonomic related activation in the small brainstem nuclei in the resting state.

Accordingly, the functional changes we found in the supratentorial CAN in our de novo PD patients, could represent a compensatory or pathologic (or both) response to the neuronal changes that, in these early stages of the disease, are expected to extensively involve the vagal nuclei complex in the brainstem and peripheral ANS constituents. However, it is possible that structural supratentorial changes have contributed to our findings. In this context, the cingulum, medial prefrontal cortex and insula are expected to be involved in inclusion bodies pathology since the clinical onset of the disease [9, 58]. Furthermore, other MRI studies based on voxel-based or tensor-based morphometry, surface-based methods or shape analysis techniques have described gray matter changes which, while with partially conflicting results, were seen to involve prefrontal, temporal, parietal and occipital cortex and in basal ganglia in early PD patients. These changes were more widespread in patients with mild cognitive impairment (MCI-PD) [32, 63–66].

### Joint rsfMRI/HRV analysis

In our study, we regressed the BOLD signal against ANS activity estimates on a single-subject level and entered the resulting correlation maps in second-level, group-wise analysis. This was done to establish a) if these correlations are significantly different from zero (i.e., to explore evidence for neural correlates of ANS activity in healthy controls or PD patients), and b) group-wise differences in these correlations (i.e., to explore possible evidence for a dysfunction in neural correlates of ANS in PD). This approach is commonly adopted in most fMRI studies (see, e.g., [67]). Specifically, it is the standard approach in both task-based studies (where the regressor is typically the task paradigm convolved with a model HRF) and resting-state which involve external regressors.

Rather than referring to a finite HRV quantifier, like entropy or spectral power, the point process analysis provides a set of “instantaneous” estimates suitable for fMRI regression analysis, without the need of interpolation [18]. Importantly, traditional interpolation techniques (linear, spline, etc.) do not account for knowledge about the physiological processes underlying the input series. Besides fitting advantages, the point-process inverse Gaussian model describes the first passage to threshold of the membrane voltages of the heart’s pacemaker cells, while the model’s autoregressive structure describes the dependence of the R-R interval lengths on the recent history of the autonomic inputs to the sino-atrial node. Also, the model’s time- varying parameters capture the dynamic character of these sino-atrial node inputs [18].

### Extensions and limitations

The main limitation of our study is the relatively small sample size and hence possible low statistical power which, however, does not reduce the validity of the significant results we observed. It may however have prevented the detection of additional ANS-related fMRI signal changes and, among those, of possible areas with positive correlations between fMRI time series and HF-HRV. In addition, in between-group analysis, we observed large effect sizes (Cohen’s d >0.8) [68]. This was as expected, since significant effects found in relative small sample sizes correspond to large effects [69, 70].

In line with previous studies [2, 52, 71], among the variety of measures that have been used to operationalize HRV, we selected HF-HRV due to its clear interpretability as a marker of purely parasympathetic autonomic outflow. On the contrary, estimates of purely sympathetic activity cannot be easily derived from HRV analysis due to the overlapping activity of both autonomic branches in the low frequency band [72, 73]. Further studies will be necessary to evaluate the possible correlations between resting state fMRI time series and others HRV metrics.

In our study, we found a network of brain areas related to parasympathetic outflow by using a correlation analysis between the BOLD signal and an external regressor in the resting state. Further studies, specifically tailored to evaluate functional and structural connectivity, will be necessary to exploit whether the CAN can be considered an intrinsic connectivity network.

Furthermore, we did not perform a quantitative evaluation of ANS function by means of specific autonomic function tests (such as the classic Ewing’s protocol [74]) that would have allowed to detect a possible subclinical cardiovascular dysfunction in our cohort of de novo PD patients. This would have been beyond the scope of the present study, whose primary aim was to evidence CAN changes in the early stages of PD and in which autonomic symptoms and signs were evaluated clinically mainly in order to exclude atypical Parkinsonisms. Further studies will be therefore necessary to evaluate the possible correlations between HRV-related fMRI changes and the degree of cardiovascular dysautonomia in de novo PD patients.

## Conclusion

In conclusion, by combining a framework for dynamical HF-HRV assessment with fMRI we have evidenced a complex network of cortical and subcortical regions modulating parasympathetic outflow at rest. Or results support the hypothesis that signs of impairment of this central network can be detectable since the early clinical stages of PD, and these changes are not due to dopaminergic replacement therapy.

## Supporting information

S1 Table Clinical features of the 14 de novo PD patients. F, female; HY, Hoehn and Yahr scale; GDS-15, Geriatric Depression Scale; NA, not available; M, male; PIGD, Postural Instability Gait Difficulty subtypes; SD, standard deviation; TD, Tremor-Dominant clinical phenotype; UPDRS, Unified Parkinson’s Disease Rating Scale.
(DOC)

S2 Table Size of automated anatomical labelling (AAL) areas and relative maximum Z score in which brain activity is significantly anti-correlated to HRV-based assessment of parasympathetic outflow (HF-HRV) in a group of 14 healthy controls [Z > 2.3 and (cluster-based corrected) cluster significance threshold of p = 0.05]. **Coordinates are expressed in MNI152 standard space. Only areas including more than 90 mm**^**3**^ **adjacent significant voxels were reported.** Ant, anterior; Inf, inferior; L, left; Mid, middle; MNI, Montreal Neurological Institute; Oper, operculum; Orb, orbital; Post, posterior; R, right; Sup, superior; Supp, supplementary; Tri, triangularis.
(DOC)

S3 Table Size of automated anatomical labelling (AAL) areas and relative maximum Z score in which brain activity is significantly anti-correlated to HRV-based assessment of parasympathetic outflow (HF-HRV) in a group of 14 de novo PD patients [Z > 2.3 and (cluster-based corrected) cluster significance threshold of p = 0.05]. **Coordinates are expressed in MNI152 standard space. Only areas including more than 90 mm**^**3**^ **adjacent significant voxels were reported.** Ant, anterior; Inf, inferior; L, left; Mid, middle; MNI, Montreal Neurological Institute; Oper, operculum; Orb, orbital; Post, posterior; R, right; Sup, superior; Tri, triangularis.
(DOC)

S4 Table Size of automated anatomical labelling (AAL) areas and relative maximum Z score where brain activity relating to HR-HRV in 14 healthy controls was significantly less than in 14 de novo PD patients [Z > 2.3 and (cluster-based corrected) cluster significance threshold of p = 0.05]. **Coordinates are expressed in MNI152 standard space. Only areas including more than 90 mm**^**3**^ **adjacent significant voxels were reported.** Ant, anterior; Inf, inferior; L, left; Mid, middle; MNI, Montreal Neurological Institute; Oper, operculum; Post, posterior; R, right; Sup, superior; Supp, supplementary.
(DOC)
