# Supplementary material for: Central modulation of parasympathetic outflow is impaired in de novo Parkinson's disease patients
Source: PLoS One. 2019 Jan 17;14(1):e0210324. doi: 10.1371/journal.pone.0210324 (PMC6336270; doi:10.1371/journal.pone.0210324)
Supplement: S1 Table — F, female; HY, Hoehn and Yahr scale; GDS-15, Geriatric Depression Scale; NA, not available; M, male; PIGD, Postural Instability Gait Difficulty subtypes; SD, standard deviation; TD, Tremor-Dominant clinical phenotype; UPDRS, Unified Parkinson’s Disease Rating Scale. (DOC) [file pone.0210324.s001.doc]

**Supplementary Table 1.** Clinical features of the 14 de novo PD patients.

| **Patient #** | **Age**  **(years)** | **Gender** | **Disease duration**  **(years)** | **HY** | **UPDRS**  **II** | **UPDRS III** | **GDS-15** | **Subtypes** |
| --- | --- | --- | --- | --- | --- | --- | --- | --- |
| 1 | 69 | F | 1.5 | 1 | 3 | 5 | 4 | TD |
| 2 | 73 | M | 1.5 | 1 | 2 | 5 | 1 | TD |
| 3 | 70 | M | 1 | 1 | 3 | 6 | 4 | TD |
| 4 | 62 | F | 1 | 1 | 4 | 8 | 9 | PIGD |
| 5 | 73 | M | 1.5 | 1.5 | 4 | 8 | 2 | TD |
| 6 | 70 | M | 1 | 1.5 | 6 | 10 | 4 | TD |
| 7 | 58 | F | 2 | 1.5 | 6 | 12 | 10 | TD |
| 8 | 69 | M | 1 | 1.5 | 6 | 8 | 5 | TD |
| 9 | 48 | M | 0.5 | 1 | 5 | 8 | 5 | TD |
| 10 | 57 | M | 0.5 | 1 | 4 | 7 | 4 | TD |
| 11 | 65 | M | 0.5 | 1 | 6 | 12 | 4 | TD |
| 12 | 78 | M | 1 | 1 | 5 | 10 | 4 | PIGD |
| 13 | 63 | M | 0.5 | 1 | 3 | 6 | NA | PIGD |
| 14 | 36 | M | 1 | 1 | 4 | 8 | 10 | TD |
| Mean  (SD) | 63.7  (11.1) |  | 1.0  (0.5) | 1.1 (0.2) | 4.4  (1.3) | 8.1  (2.3) | 5.1  (2.8) |  |

I

F, female; HY, Hoehn and Yahr scale; GDS-15, Geriatric Depression Scale; NA, not available; M, male; PIGD, Postural Instability Gait Difficulty subtypes; SD, standard deviation; TD, Tremor-Dominant clinical phenotype; UPDRS, Unified Parkinson’s Disease Rating Scale.
