# Supplementary material for: Central modulation of parasympathetic outflow is impaired in de novo Parkinson's disease patients
Source: PLoS One. 2019 Jan 17;14(1):e0210324. doi: 10.1371/journal.pone.0210324 (PMC6336270; doi:10.1371/journal.pone.0210324)
Supplement: S2 Table — Coordinates are expressed in MNI152 standard space. Only areas including more than 90 mm3 adjacent significant voxels were reported. Ant, anterior; Inf, inferior; L, left; Mid, middle; MNI, Montreal Neurological Institute; Oper, operculum; Orb, orbital; Post, posterior; R, right; Sup, superior; Supp, supplementary; Tri, triangularis. (DOC) [file pone.0210324.s002.doc]

***Supplementary Table 2.*** *Size of Automated Anatomical Labelling (AAL) areas and relative maximum Z score in which brain activity is significantly anti-correlated to HRV-based assessment of parasympathetic outflow (HF-HRV) in a group of 14 healthy controls [Z > 2.3 and (cluster-based corrected) cluster significance threshold of p = 0.05]. Coordinates are expressed in MNI152 standard space. Only areas including more than 90 mm^3^ adjacent significant voxels were reported.*

| AAL anatomical area | Size  (mm^3^) | Z max | Z max  X  (mm) | Z max  Y  (mm) | Z max  Z  (mm) |
| --- | --- | --- | --- | --- | --- |
| ***Cerebral areas*** |  |  |  |  |  |
| *Cortical* |  |  |  |  |  |
| R Cingulum Mid | 11711 | 3.3 | 9 | 16 | 34 |
| L Cingulum Mid | 9835 | 3.3 | 0 | 11 | 34 |
| R Postcentral | 9539 | 3.2 | 19 | -36 | 68 |
| R Temporal Sup | 9487 | 3.5 | 60 | -18 | 8 |
| R Fusiform | 8556 | 3.4 | 28 | -61 | -11 |
| R Insula | 8367 | 3.6 | 31 | -23 | 14 |
| R Lingual | 8206 | 3.6 | 15 | -77 | -7 |
| R Precentral | 8024 | 3.2 | 11 | -28 | 77 |
| L Paracentral Lobule | 7004 | 3.9 | -2 | -21 | 77 |
| R Supp Motor Area | 6861 | 3.4 | 16 | 8 | 67 |
| L Postcentral | 6855 | 3.2 | -19 | -36 | 73 |
| R Calcarine | 6190 | 3.4 | 10 | -87 | 2 |
| R Frontal Sup | 6034 | 3.5 | 19 | 8 | 67 |
| L Precuneus | 5730 | 3.1 | -6 | -43 | 58 |
| R Paracentral Lobule | 5191 | 3.7 | 2 | -20 | 77 |
| R Angular | 4946 | 2.9 | 45 | -55 | 40 |
| L Temporal Sup | 4651 | 3.3 | -46 | -23 | 9 |
| R Precuneus | 4390 | 3.2 | 25 | -51 | 3 |
| R Parietal Inf | 4339 | 2.9 | 46 | -53 | 44 |
| R Temporal Mid | 4181 | 3.3 | 68 | -29 | 0 |
| L Parietal Inf | 4078 | 3 | -32 | -47 | 49 |
| R Temporal Inf | 3916 | 3.1 | 59 | -22 | -21 |
| L Supp Motor Area | 3847 | 3.2 | -2 | -21 | 51 |
| R SupraMarginal | 3773 | 3.1 | 52 | -33 | 24 |
| R Cingulum Ant | 3765 | 3.2 | 11 | 20 | 29 |
| L Fusiform | 3709 | 3.6 | -44 | -62 | -20 |
| R Frontal Inf Tri | 3529 | 3.1 | 41 | 26 | 3 |
| L Calcarine | 3210 | 3.2 | 4 | -86 | 0 |
| R Frontal Mid | 3174 | 2.9 | 27 | 37 | 24 |
| R Temporal Pole Sup | 2881 | 3.3 | 50 | 16 | -11 |
| L Frontal Sup Medial | 2752 | 3.3 | -3 | 68 | 4 |
| R Frontal Sup Medial | 2745 | 3.1 | 1 | 68 | 8 |
| R Rolandic Oper | 2724 | 3.4 | 52 | -18 | 11 |
| R Frontal Inf Oper | 2537 | 3.1 | 56 | 18 | -1 |
| L Occipital Sup | 2412 | 3.1 | -10 | -96 | 12 |
| R Cuneus | 2329 | 3.3 | 14 | -91 | 8 |
| L Insula | 2306 | 3.3 | -32 | -18 | 20 |
| R Frontal Sup Orb | 2201 | 3 | 22 | 33 | -24 |
| L Rolandic Oper | 2106 | 3.3 | -40 | -34 | 16 |
| R Occipital Sup | 1916 | 3.3 | 16 | -92 | 6 |
| R Parietal Sup | 1892 | 2.8 | 19 | -42 | 69 |
| L Lingual | 1880 | 3.1 | -3 | -77 | -1 |
| R Frontal Mid Orb | 1838 | 3 | 38 | 55 | -16 |
| L Cingulum Ant | 1795 | 3 | -2 | 10 | 31 |
| R Frontal Inf Orb | 1734 | 3.3 | 48 | 19 | -8 |
| L Frontal Sup Orb | 1712 | 3 | -28 | 63 | -1 |
| L Occipital Mid | 1479 | 2.9 | -11 | -97 | 3 |
| R Heschl | 1467 | 3.6 | 33 | -28 | 13 |
| R ParaHippocampal | 1385 | 3.3 | 21 | -20 | -25 |
| R Hippocampus | 1342 | 3.3 | 25 | -33 | 9 |
| R Occipital Inf | 1257 | 3.5 | 33 | -73 | -6 |
| L Temporal Mid | 1256 | 2.9 | -65 | -33 | 1 |
| L Parietal Sup | 1048 | 2.9 | -31 | -48 | 50 |
| L Heschl | 1048 | 3.2 | -46 | -20 | 10 |
| L Frontal Med Orb | 968 | 3.1 | -5 | 67 | -1 |
| L Precentral | 904 | 3.1 | -19 | -20 | 73 |
| R Frontal Med Orb | 884 | 2.9 | 12 | 69 | -3 |
| L Frontal Sup | 823 | 3.1 | -28 | 65 | 1 |
| L Occipital Inf | 747 | 3.2 | -48 | -63 | -17 |
| L Cingulum Post | 671 | 2.8 | -3 | -32 | 28 |
| L Hippocampus | 631 | 2.7 | -24 | -25 | -12 |
| L Cuneus | 604 | 3.1 | -9 | -96 | 13 |
| L SupraMarginal | 590 | 2.7 | -60 | -23 | 14 |
| R Rectus | 580 | 2.7 | 2 | 46 | -23 |
| L Temporal Inf | 561 | 3.1 | -49 | -56 | -23 |
| L ParaHippocampal | 463 | 2.6 | -20 | -41 | -4 |
| R Temporal Pole Mid | 355 | 2.6 | 47 | 18 | -27 |
| R Cingulum Post | 186 | 2.7 | 2 | -40 | 26 |
| L Frontal Mid | 134 | 3 | -28 | 65 | 2 |
| L Frontal Mid Orb | 125 | 2.6 | -31 | 61 | -6 |
| *Subcortical* |  |  |  |  |  |
| L Thalamus | 3725 | 2.9 | -15 | -14 | 19 |
| R Putamen | 2808 | 3 | 29 | -17 | 8 |
| R Thalamus | 2465 | 3.5 | 22 | -27 | 12 |
| R Caudate | 1015 | 2.7 | 20 | -23 | 20 |
| L Caudate | 877 | 3.1 | -17 | -9 | 21 |
| L Putamen | 790 | 3.1 | -29 | -15 | 13 |
| R Pallidum | 508 | 2.7 | 21 | 4 | -6 |
| L Pallidum | 125 | 2.5 | -11 | 1 | 1 |
| ***Cerebellar areas*** |  |  |  |  |  |
| R Cerebelum 6 | 9715 | 3.3 | 26 | -59 | -17 |
| L Cerebelum Crus1 | 7958 | 3.6 | -35 | -75 | -26 |
| R Cerebelum Crus1 | 7546 | 3.5 | 44 | -73 | -24 |
| L Cerebelum 6 | 6688 | 3.6 | -31 | -59 | -20 |
| L Cerebelum 4 5 | 4988 | 3.4 | -30 | -39 | -28 |
| R Cerebelum 8 | 4788 | 3.1 | 30 | -55 | -53 |
| R Cerebelum 4 5 | 4411 | 3.2 | 14 | -46 | -17 |
| L Cerebelum 8 | 3671 | 3 | -34 | -44 | -44 |
| L Cerebelum 9 | 3012 | 3.1 | -11 | -49 | -47 |
| Vermis 4 5 | 2486 | 3.2 | -3 | -59 | -9 |
| Vermis 6 | 2151 | 3.2 | 2 | -65 | -9 |
| L Cerebelum Crus2 | 2032 | 2.8 | -28 | -71 | -39 |
| R Cerebelum 9 | 1617 | 2.6 | 13 | -44 | -46 |
| R Cerebelum Crus2 | 1458 | 3 | 21 | -88 | -30 |
| Vermis 7 | 1211 | 2.8 | 1 | -68 | -28 |
| R Cerebelum 3 | 1032 | 3.2 | 17 | -24 | -24 |
| Vermis 8 | 845 | 2.9 | 6 | -71 | -40 |
| L Cerebelum 7b | 693 | 2.8 | -37 | -37 | -37 |
| Vermis_3 | 447 | 2.9 | 5 | -43 | -10 |
| R Cerebelum 7b | 339 | 3 | 7 | -74 | -44 |
| R Cerebelum 10 | 211 | 3 | 24 | -37 | -46 |
| L Cerebelum 3 | 112 | 2.6 | -5 | -43 | -13 |

Ant, anterior; Inf, inferior; L, left; Mid, middle; MNI, Montreal Neurological Institute; Oper, operculum; Orb, orbital; Post, posterior; R, right; Sup, superior; Supp, supplementary; Tri, triangularis.
