# Supplementary material for: Central modulation of parasympathetic outflow is impaired in de novo Parkinson's disease patients
Source: PLoS One. 2019 Jan 17;14(1):e0210324. doi: 10.1371/journal.pone.0210324 (PMC6336270; doi:10.1371/journal.pone.0210324)
Supplement: S3 Table — Coordinates are expressed in MNI152 standard space. Only areas including more than 90 mm3 adjacent significant voxels were reported. Ant, anterior; Inf, inferior; L, left; Mid, middle; MNI, Montreal Neurological Institute; Oper, operculum; Orb, orbital; Post, posterior; R, right; Sup, superior; Tri, triangularis. (DOC) [file pone.0210324.s003.doc]

**Supplementary Table 3.** Size of Automated Anatomical Labelling (AAL) areas and relative maximum Z score in which brain activity is significantly anti-correlated to HRV-based assessment of parasympathetic outflow (HF-HRV) in a group of 14 de novo PD patients [Z > 2.3 and (cluster-based corrected) cluster significance threshold of p = 0.05]. Coordinates are expressed in MNI152 standard space. Only areas including more than 90 mm^3^ adjacent significant voxels were reported.

| AAL anatomical area | Size  (mm^3^) | Z max | Z max  X  (mm) | Z max  Y  (mm) | Z max  Z  (mm) |
| --- | --- | --- | --- | --- | --- |
| ***Cerebral areas*** |  |  |  |  |  |
| *Cortical* |  |  |  |  |  |
| R Temporal Mid | 7088 | 3.2 | 66 | -11 | -15 |
| R Frontal Inf Tri | 4730 | 3.1 | 48 | 32 | 7 |
| R Frontal Inf Orb | 4331 | 3 | 49 | 44 | -3 |
| R Temporal Sup | 4020 | 3.1 | 67 | -11 | -10 |
| R Temporal Inf | 3230 | 2.9 | 56 | -45 | -9 |
| R Insula | 1870 | 3 | 30 | 20 | -18 |
| R Angular | 1570 | 3 | 42 | -56 | 25 |
| R Frontal Inf Oper | 1148 | 2.8 | 62 | 15 | 10 |
| R Temporal Pole Sup | 1018 | 2.8 | 31 | 21 | -25 |
| R Frontal Mid Orb | 796 | 2.9 | 50 | 47 | -7 |
| R Postcentral | 600 | 3 | 67 | -5 | 17 |
| R Rolandic Oper | 441 | 2.9 | 66 | -6 | 13 |
| R Olfactory | 365 | 2.8 | 23 | 16 | -17 |
| R Frontal Mid | 281 | 2.7 | 49 | 45 | 2 |
| R SupraMarginal | 235 | 2.5 | 68 | -40 | 24 |
| R Occipital Inf | 196 | 2.6 | 51 | -64 | -13 |
| R Temporal Pole Mid | 102 | 2.5 | 41 | 24 | -38 |
| *Subcortical* |  |  |  |  |  |
| R Caudate | 953 | 2.9 | 12 | 4 | 12 |
| R Putamen | 856 | 2.7 | 18 | 12 | -8 |
| R Pallidum | 160 | 2.6 | 16 | 8 | -5 |

Ant, anterior; Inf, inferior; L, left; Mid, middle; MNI, Montreal Neurological Institute; Oper, operculum; Orb, orbital; Post, posterior; R, right; Sup, superior; Tri, triangularis.
