# Supplementary material for: Central modulation of parasympathetic outflow is impaired in de novo Parkinson's disease patients
Source: PLoS One. 2019 Jan 17;14(1):e0210324. doi: 10.1371/journal.pone.0210324 (PMC6336270; doi:10.1371/journal.pone.0210324)
Supplement: S4 Table — Coordinates are expressed in MNI152 standard space. Only areas including more than 90 mm3 adjacent significant voxels were reported. Ant, anterior; Inf, inferior; L, left; Mid, middle; MNI, Montreal Neurological Institute; Oper, operculum; Post, posterior; R, right; Sup, superior; Supp, supplementary. (DOC) [file pone.0210324.s004.doc]

**Supplementary Table 4.** Size of Automated Anatomical Labelling (AAL) areas and relative maximum Z score where brain activity relating to HR-HRV in 14 healthy controls was significantly less than in 14 de novo PD patients [Z > 2.3 and (cluster-based corrected) cluster significance threshold of p = 0.05]. Coordinates are expressed in MNI152 standard space. Only areas including more than 90 mm^3^ adjacent significant voxels were reported.

| AAL anatomical area | Size  (mm^3^) | Z max | Z max  X  (mm) | Z max  Y  (mm) | Z max  Z  (mm) |
| --- | --- | --- | --- | --- | --- |
| ***Cerebral areas*** |  |  |  |  |  |
| *Cortical* |  |  |  |  |  |
| L Cingulum Mid | 5501 | 3.2 | 0 | -23 | 51 |
| L Paracentral Lobule | 5129 | 3.8 | -2 | -18 | 77 |
| R Cingulum Mid | 4913 | 3.2 | 1 | -23 | 51 |
| R Precentral | 4302 | 3.3 | 24 | -19 | 72 |
| R Paracentral Lobule | 4040 | 3.6 | 1 | -21 | 76 |
| R Supp Motor Area | 3362 | 3.5 | 1 | -19 | 75 |
| R Postcentral | 3019 | 3.2 | 17 | -35 | 68 |
| L Postcentral | 2838 | 2.9 | -20 | -26 | 70 |
| L Precuneus | 2600 | 3.1 | -7 | -46 | 57 |
| R Frontal Sup | 2370 | 3.1 | 16 | -8 | 72 |
| R Lingual | 1717 | 3.2 | 16 | -51 | -11 |
| L Supp Motor Area | 1327 | 3.1 | -2 | -21 | 51 |
| L Parietal Inf | 1268 | 2.9 | -36 | -41 | 49 |
| L Calcarine | 1241 | 2.7 | 2 | -82 | -5 |
| L Lingual | 904 | 3 | -24 | -59 | -13 |
| L Fusiform | 712 | 3.2 | -24 | -66 | -16 |
| R Rolandic Oper | 542 | 3 | 37 | -30 | 21 |
| L Precentral | 542 | 3 | -19 | -21 | 73 |
| R Insula | 463 | 3 | 37 | -29 | 22 |
| R Temporal Sup | 306 | 3.2 | 34 | -29 | 11 |
| R Heschl | 301 | 3.5 | 33 | -29 | 12 |
| R Precuneus | 294 | 2.7 | 0 | -42 | 59 |
| R SupraMarginal | 264 | 2.8 | 43 | -29 | 24 |
| R Fusiform | 196 | 3 | 18 | -49 | -13 |
| R Hippocampus | 143 | 3.2 | 26 | -33 | 8 |
| L Cingulum Ant | 101 | 2.8 | -5 | -1 | 31 |
| L Cingulum Post | 92 | 2.7 | -5 | -31 | 33 |
| *Subcortical* |  |  |  |  |  |
| R Putamen | 357 | 2.9 | 28 | -10 | 14 |
| ***Cerebellar areas*** |  |  |  |  |  |
| L Cerebelum 6 | 6106 | 3.4 | -23 | -57 | -20 |
| L Cerebelum 4 5 | 3942 | 3.4 | -19 | -53 | -20 |
| R Cerebelum 4 5 | 2792 | 3.3 | 15 | -49 | -13 |
| Vermis 6 | 1669 | 3.1 | 3 | -65 | -8 |
| Vermis 4 5 | 1645 | 3.2 | -3 | -58 | -6 |
| L Cerebelum Crus1 | 1552 | 3.3 | -34 | -79 | -21 |
| R Cerebelum 6 | 1456 | 3.1 | 11 | -81 | -20 |
| R Cerebelum Crus1 | 452 | 3.2 | 11 | -82 | -21 |
| Vermis 7 | 449 | 2.7 | 1 | -74 | -20 |
| Vermis 3 | 205 | 3.1 | 6 | -43 | -8 |
| Cerebelum 10 L | 183 | 3.1 | -21 | -34 | -39 |
| Vermis 8 | 178 | 2.6 | 0 | -67 | -29 |
| R Cerebelum 3 | 141 | 3 | 7 | -41 | -9 |

Ant, anterior; Inf, inferior; L, left; Mid, middle; MNI, Montreal Neurological Institute; Oper, operculum; Post, posterior; R, right; Sup, superior; Supp, supplementary.
